# Apical Extrusion: Is It an Inherent Occurrence During Every Endodontic Treatment?

**DOI:** 10.7759/cureus.45211

**Published:** 2023-09-14

**Authors:** Kaoutar Laslami, Salma Khaldoune, Aly Sy, Sofia DROURI, Imane Benkiran

**Affiliations:** 1 Department of Conservative Dentistry and Endodontics, Faculty of Dental Medicine, University Hassan II, Casablanca, MAR

**Keywords:** flare up, prevention, irrigation, instrumentation, root canal preparation, bacterial, debris, apical extrusion

## Abstract

The aim of this literature review was to analyze all available scientific data on apical extrusion and to identify and associate the possible factors influencing the occurrence of apical extrusion, ranging from the choice of the canal shaping system, the irrigation technique, to the choice of diameter and the apical limit of preparation. A bibliographic search for relevant articles on apical extrusion of debris, irrigants, and bacteria was performed using the following databases: PubMed, Cochrane Library, and ScienceDirect. This search identified articles published between 2010 and 2023 in two languages (French and English). After selective sorting, 25 relevant documents were included. All the studies retained clearly agree on the inherent nature of apical extrusion during endodontic treatment. At the same time, we also understand that the amount of extrusion can be influenced by a number of parameters throughout endodontic therapy. According to this review, despite the undeniable nature of apical extrusion during endodontic therapy, studies with relatable experimental models that approach in vivo conditions are required to establish exploitable conclusions regarding apical extrusion and its prevention.

## Introduction and background

The goal of every endodontic treatment is to prevent or eliminate inflammatory pulp and periapical pathologies. Achieving this objective requires the elimination of bacteria and toxins that have colonized the endodontic system, as well as the debris that serves as their nutrient, all while respecting the integrity of the periapical tissues [1]. Therefore, bacterial extrusion beyond the apex could jeopardize the treatment's success. These apically extruded bacteria are linked to acute inflammatory responses in periapical tissues, which clinically manifest as postoperative pain and/or swelling. This phenomenon, also known as an endodontic flare-up, is often associated with the failure of endodontic therapy. While this undesirable episode is infrequent and temporary, it can nonetheless confuse the patient [2].

Although bacteria are the primary cause of inducing inflammation, this inflammatory outbreak is actually multifactorial. Mechanical and chemical factors also contribute to it [3]. Various types of physical or chemical irritants, such as drugs, irrigating solutions, or chemically modified tissue proteins, can disrupt the established equilibrium between the bacterial flora present in the periapex and the host's defenses, potentially triggering an acute periapical reaction [2].

On the other hand, preventing all types of periapical tissue damage and irritation is a major clinical responsibility for every practitioner. To prevent this dreaded postoperative complication, we have focused on one of its main etiologies: apical extrusion of debris. Therefore, the aim of this literature review is to provide a comprehensive summary of apical debris extrusion and to explore all aspects of this phenomenon during endodontic procedures. This includes a particular focus on factors that influence it, such as the choice of canal shaping system, irrigation technique, choice of diameter, and apical limit of preparation.

## Review

Methods

A search was conducted on PubMed, Cochrane Library, and Science Direct using the following keywords: apical extrusion, debris, bacterial, root canal preparation, instrumentation, irrigation, prevention, and flare-up. These terms were used either individually or in combination. The scope of the publications analyzed was limited to articles published between 2010 and 2023 that specifically dealt with the apical extrusion of debris, bacteria, and irrigating solutions. Publications that evaluated extrusion in temporary teeth, extrusion in immature permanent teeth, and extrusion of paste and/or filling material were excluded. Additionally, publications in languages other than French and English were not considered. Further articles were included from the bibliographic references of the publications initially analyzed.

Results

**Figure 1:**
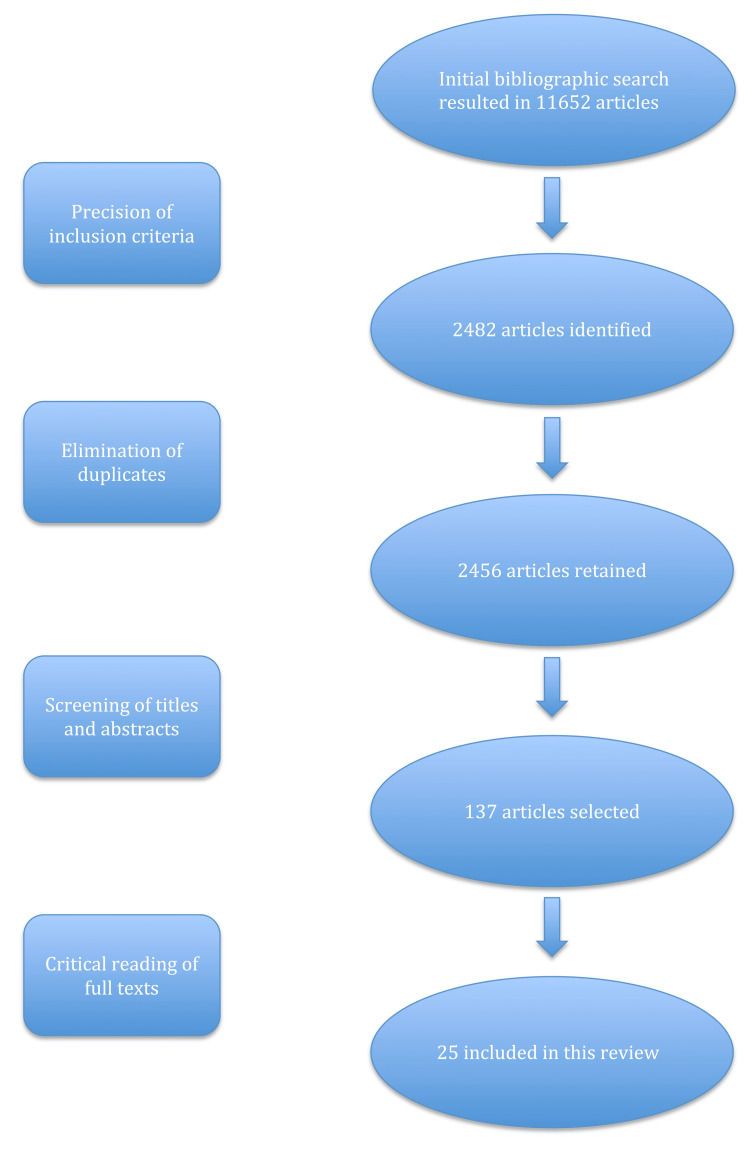
Flow diagram for the data search

The initial screening elicited 11,652 studies. However, after implementing the inclusion and exclusion criteria, selective sorting was carried out. The selection was first made according to the titles and abstracts which revealed 137 studies. Then a complete reading of the full texts of articles allowed to remove all studies with poor methodological quality or insufficient data. Finally, this bibliographic search included 25 relevant documents relating to our subject. A diagram describing the data search is shown in Figure 1, followed by a table summarizing the selected studies (Table 1).

**Table 1:** Summary table of selected studies

Study	Method	Results
A critical analysis of research methods and experimental models to study apical extrusion of debris and irrigants. Tanalp (2022) [4].	–This review analyzed experimental models and research methods studying apical extrusion of debris and irrigants, by conducting a search in both PubMed and Scopus. –144 relevant articles were selected from 1968 until 2021. –The search was conducted by inserting the following keywords: ‘apical extrusion and debris’ or ‘extrusion, debris, and dentistry’ or ‘extrusion, irrigant, and endodontics’ or ‘extrusion, irrigation, and endodontics’. –This review summarized each methodology by presenting a general description and making a critical analysis of their advantages and drawbacks.	–All methodologies discussed in this review have their own advantages and drawbacks. –Quantitative evaluation of debris and irrigants only serves as a numerical representation, which neglects some biological parameters like bacterial virulence and host response. –A representative model of clinical conditions should be developed to evaluate the phenomenon in all its circumstances, in order to better understand this clinically relevant occurrence.
Influence of apical enlargement in cleaning and extrusion in canals with mild and moderate curvatures. Borges et al. (2011) [5].	–Evaluation of the cleaning and the extrusion in the apical region of the root canal by comparing two groups of different maxillary lateral incisors: (1) Group 1: with curvature angles ≤10° (mild curvature), (2) Group 2: with curvature between 11° to 25° (moderate curvature). –Each group was subdivided in two: to be prepared with rotary instrumentation with different apical diameters 30.02 vs. 45.02. –This study used optical microscopy and morphometric analysis.	–No statistically significant differences were observed among all groups concerning canal cleaning (irrespective of canal curvature and apical diameter). –Considering the amount of extruded material, canals with mild curvature prepared with 45.02 instruments showed significantly higher values than those of the other groups, which were similar among themselves.
Apical extrusion of Enterococcus faecalis in different canal geometries during the use of nickel-titanium systems with different motion types. Aydin et al. (2017) [6].	–Comparison of three groups of teeth with different canal geometries: (1) Mandibular incisors: narrow straight canals, (2) Mandibular premolars: large straight canals, (3) Maxillary 1st molars (mesiobuccal root): curved canals. –Each group is divided into subgroups of canal shaping: (1) Reciproc, (2) OneShape, (3) Twisted File Adaptive. –Evaluation method: bacterial extrusion (using Enterococcus faecalis suspension).	–Apical extrusion of E. faecalis is statistically equal in all subgroups. –Canal morphology has no influence on the degree of extrusion.
Influence of working length and apical preparation size on apical bacterial extrusion during reciprocating instrumentation. Teixeira et al. (2015) [7].	–Quantitative comparison of bacterial extrusion in four groups: (1) Group R25/0: Reciproc 25 taper 08 to working length at the apical foramen; (2) Group R25/1: Reciproc 25 taper 08 to working length at 1mm short of the apical foramen; (3) Group R40/0: Reciproc 40 taper 06 to working length at the apical foramen; (4) Group R40/1: Reciproc 40 taper 06 to working length at 1mm short of the apical foramen.	–No significant difference was found in the number of colony-forming units among all experimental groups.
Extrusion of debris from curved root canals instrumented up to different working lengths using different reciprocating systems. Mendonça de Moura et al. (2019) [8].	–Quantitative evaluation of debris in Eppendorf for four groups: (mandibular 1st molar with mesiobuccal canal curvature between 10° and 20°): (1) Group 1: Reciproc to working length at the apical foramen; (2) Group 2: Reciproc to working length at 1mm short of the apical foramen; (3) Groupe 3: WaveOne to working length at the apical foramen; (4) Group 4: WaveOne to working length at 1mm short of the apical foramen.	–No statistically significant difference was observed between the four experimental groups regarding the amount of debris extruded.
Effect of root canal dimensions, injection rate, and needle design on the apical extrusion of an irrigant: an in vitro study. Chang et al. (2015) [9].	–Quantitative evaluation of irrigants in artificial canals (made in acrylic blocks) prepared to a standard size of ISO (25, 30, or 40) with the same taper (0.06). –Irrigant solution is delivered by two needle types (notched end/ side vented) and to four different injection rates (50/100/200/300 μl/s).	–Irrigant extrusion concerned all groups. –Increasing the injection rate beyond 200 μl/s increases the extrusion risk. –Using a side-vented needle decreases the extrusion risk. –Increasing the canal size to ISO40 decreases the extrusion risk.
Influence of apical preparation size and working length on debris extrusion. Silva et al. (2016) [10].	–Quantitative evaluation of debris for four groups (mandibular incisor) with same curvature (<10°): (1) Group 1: Reciproc 25 taper 08 to working length at the apical foramen; (2) Group 2: Reciproc 40 taper 06 to working length; (3) Group 3: Reciproc 25 taper 08 to working length at 1mm short of the apical foramen; (4) Group 4: Reciproc 40 taper 06 to working length at 1mm short of the apical foramen.	–No significant difference was found between the different working lengths in regard to debris extrusion. –No significant difference was found between the two apical diameters regarding debris extrusion.
Can kinematics, file diameter, and PUI influence intracanal decontamination and apical bacterial extrusion? Cuellar et al. (2021) [11].	–This study recorded two types of extrusion: debris and bacterial (E. feacalis suspension). –It explored three parameters: kinematics, file diameter (25/35), and irrigation technique (conventional irrigation, passive ultrasonic irrigation).	–The same amount of extruded debris and bacteria was found in all the tested systems (regarding different file diameters and kinematics). –Passive ultrasonic irrigation resulted in greater extrusion of contaminated dentinal debris.
Apically extruded debris produced during glide path preparation using R-Pilot, WaveOne Gold Glider, and ProGlider in curved root canals. Keskin et al. (2020) [12].	–Comparing apical extrusion produced in different glide path preparations. –20 canals per group were used to evaluate: the manual glide path by K-files, ProGlider, R-pilot, and WaveOne Gold Glider.	–All groups caused apical extrusion with manual preparation producing the highest amount. –There were no significant differences among the extruded debris between ProGlider, R-pilot, and WaveOne Gold Glider.
Influence of instruments used in root canal preparation on amount of apically extruded debris. Karataş et al. (2016) [13].	–Quantitative comparison of debris extrusion in four groups to evaluate heat-treated systems: (1) Group 1: Protaper Universal; (2) Group 2: Protaper Gold; (3) Group 3: WaveOne; (4) Group 4: WaveOne Gold.	–All systems caused apical extrusion of debris. –The amount of extrusion is reduced for heat-treated systems.
The influence of two reciprocating single-file and two rotary-file systems on the apical extrusion of debris and its biological relationship with symptomatic apical periodontitis. Caviedes-Bucheli et al. (2016) [14].	–This systematic review and meta-analysis focused on the influence of the number of files used during canal preparation on apical extrusion and its biological relationship with the occurrence of symptomatic apical periodontitis. –Literature research was conducted in Medline, ISI Web of Science, and Cochrane databases. –The systematic review included nine laboratory studies and two in vivo studies. –The meta-analysis included four additional in vitro studies.	–All root canal instrumentation techniques caused apical extrusion of debris in laboratory studies or expression of neuropeptides in vivo. –The inflammatory reaction is not influenced by the number of files.
Pain after root canal treatment with different instruments. Sun et al. (2018) [15].	–This study aimed to compare the incidence and intensity of postoperative pain after canal treatment using manual rotary and reciprocating instruments. –Literature research was conducted in PubMed, EMBASE, Web of Science, and Cochrane Library. –Studies included in the systematic review were 17, and nine of them were used in the meta-analysis.	–Rotary instruments generated a lower incidence and intensity of post-operative pain in patients than did hand file instruments. –Using rotary multiple rotary-file systems contributed to a lower incidence of postoperative pain than did the use of reciprocating systems.
A comparison of apical bacterial extrusion in the manual, ProTaper rotary, and one-shape rotary instrumentation techniques. Mittal et al. (2015) [16].	–Evaluation of bacterial extrusion in 42 mandibular premolars divided into three groups using different instrumentation techniques: (1) Manual group using K-files; (2) Protaper group; (3) OneShape group.	–All instruments generated bacterial extrusion. –Manual step-back technique resulted in significantly more extrusion than the engine-driven systems. –Protaper rotary extruded significantly more bacteria than the OneShape rotary system.
Assessment of apically extruded debris and irrigant produced by different nickel-titanium instrument systems. Küçükyilmaz et al. (2015) [17].	–Comparison of preparation time and the amounts of apically extruded debris and irrigants using three different NiTi systems, in 45 mandibular premolars divided into three groups: (1) Protaper: multiple files in continuous rotation; (2) Reciproc: single file in reciprocity; (3) OneShape: single file in continuous rotation.	–For extrusion of debris and irrigants: no statistically relevant difference was found between all groups. –For the preparation time: (1) No difference between single file instrumentation systems. (2) The difference was significant between single-file and multiple-file instrumentation.
The effect of different kinematics on apical debris extrusion with a single-file system. Al Omari et al. (2023) [18].	–Evaluation of extruded debris caused by different motions in 50 mandibular first molars randomized in five groups: (1) Optimize Torque Reverse (OTR); (2) Twisted File Adaptive motion (TFA); (3) Continuous rotation (CR); (4) Reciprocation motion (REC); (5) Jeni motion (Jeni).	–All motions extruded debris apically. –Jeni motion produced significantly less apically extruded debris compared with TFA, REC, and CR and no significant difference emerged with OTR.
Incidence of postoperative pain after using single continuous, single reciprocating, and full sequence continuous rotary file system: a prospective randomized clinical trial Kumar et al. (2023) [19].	–Patients were assigned according to the system used: Protaper Next, OneShape, or WaveOne Gold. –Each practitioner included in this study prepared 21 teeth and randomly selected seven per instrument. –Initial pain was recorded and then postoperative pain at 24, 48, 72h, and 7 day post endodontic treatment.	–No statistically relevant difference among Protaper Next, OneShape, and WaveOne Gold regarding the incidence of postoperative pain.
Bacteria and hard tissue debris extrusion and intracanal bacterial reduction promoted by XP-endo Shaper and Reciproc Instruments. Alves et al. (2018) [20].	–Evaluation of bacterial and hard tissue extruded apically with the use of Reciproc and XP-endo Shaper. –Maxillary molars were distributed into two groups depending on the used system.	–Both instruments produced a similar volume of extruded debris, extruded bacteria counts were significantly lower with Reciproc than the XP-endo Shaper.
Effect of mode of rotation on apical extrusion of debris with four different single-file endodontic instrumentation systems: Systematic review and meta-analysis Ahmad et al. (2022) [21].	–Comparing apical extrusion produced by single file systems in full rotational motion (NeoNiTi and/ OneShape) versus reciprocating motion (WaveOne and/ Reciproc). –Literature research was conducted in Medline, Embase, and Web of Science, the top three specialty journals (Journal of Endodontics, International Endodontic Journal, and Australian Endodontic Journal) for the last 10 years were also searched. Gray literature was investigated using Open Grey and Google Scholar to explore unpublished studies. –A total of eight studies were included in this review.	–Single-file reciprocating systems tend to cause more extrusion of debris than single-file rotary systems.
Evaluation of apically extruded debris using positive and negative pressure irrigation systems in association with different irrigants. Barbosa-Ribeiro et al. (2018) [22].	–Evaluation of extruded debris using two irrigation techniques each one associated with four different irrigants. –1stcomparison between the two irrigation techniques: (1) positive pressure: Conventional Needle Irrigation (CNI), (2) negative pressure: Endovac system. –2ndcomparison between four irrigants: (1) 6% NaOCl, (2) 2% Chlorhexidine gel+ Saline Solution, (3) 2% Chlorhexidine solution, (4) Saline Solution.	–Apical extrusion concerned all groups. –Endovac resulted in lower levels of debris extrusion than conventional needle irrigation. –No differences were observed regarding the irrigant when Endovac was used. –When CNI was used, chlorhexidine gel + saline solution was associated with lower debris extrusion.
Effect of needle insertion depth and root canal curvature on irrigant extrusion. Psimma et al. (2013) [23].	–Evaluate the effect of needle type and insertion depth, apical preparation size, and root canal curvature on irrigant extrusion in two groups: (1) Group A: 16 straight root canals and (2) Group B: 16 moderately curved root canals. –Preparation sizes were 25 or 35 both with 06 taper, using rotary NiTi instruments. –Needle types were either open-ended or closed-ended positioned at 1, 3, or 5 mm short of working length.	–Root canal curvature did not have a significant effect on irrigant extrusion. –The open-ended needle caused significantly more irrigant extrusion than the closed-ended one. –Needle wedging increased extrusion, especially when using an open-ended needle. –Irrigant extrusion decreased as needles moved away from working length or when the apical size was increased.
Weight of apically extruded debris following the use of two canal instrumentation techniques and two designs of irrigation needles. Yeter et al. (2013) [24].	–Comparison of debris extruded in 40 mandibular premolars: (1) Group 1a: Hand instrumentation with an open-ended needle, (2) Group 1b: Hand instrumentation with a two side-port needle, (3) Group 2a: Revo-S NiTi instrument preparation with an open-ended needle, (4) Group 2b: Revo-S NiTi instrument preparation with a two side-port needle.	–Open-ended needles were associated with significantly more debris than side-vented needles. –No statistically significant difference was observed between K-files and the RevoS system.
Does apical negative pressure prevent the apical extrusion of debris and irrigant compared with conventional irrigation? A systematic review and meta-analysis by Romualdo et al. (2017) [25].	–Evaluation of irrigation by apical negative pressure in preventing apical extrusion in comparison to needle irrigation. –Literature research performed using: Medline, Web of Science, Bireme, and Scopus databases. –Studies included in the meta-analysis were four.	–Apical negative pressure prevents the apical extrusion of irrigants with no evidence if this type of irrigation can prevent the extrusion of debris.
Comparison of sodium hypochlorite extrusion by five irrigation systems using an artificial root socket model and a quantitative chemical method. Azim et al. (2018) [26].	–Evaluation of NaOCl extrusion during three irrigant activation techniques: (1) mechanical activation: XP-EndoFinisher, (2) sonic activation: EndoActivator, (3) laser activation: Photo-Induced Photoacoustic Streaming (PIPS). –Compared to two techniques with no activation: (1) positive pressure: standard needle irrigation (SNI), (2) apical negative pressure: Endovac.	–All techniques were accompanied by extrusion. –The Endovac registered the least extrusion. –No significantly relevant difference was observed between standard needle irrigation, sonic activation, and mechanical activation. –PIPS laser generated the largest extrusion volume compared to other irrigation techniques.
Evaluation of 4 different irrigating systems for apical extrusion of sodium hypochlorite. Yost et al. (2015) [27].	–Evaluation of apical extrusion of NaOCl using four irrigating techniques in an in vitro color-changing pH-sensitive gel model. –Pre-instrumentation and post-instrumentation photographs were taken to be analyzed numerically by ImageJ software. –The tested techniques were: (1) apical negative pressure (Endovac), (2) sonic agitation (EndoActivator), (3) side-vented needle (Max-i-Probe), (4) laser: photon-induced photoacoustic streaming (PIPS) in two settings 10 mJ and 20 mJ.	–There were no significant differences between Endovac and EndoActivator. –The Endovac and EndoActivator groups caused significantly less extrusion than PIPS irrigation. –The Endovac system showed significantly less extrusion potential than PIPS irrigation and the Max-i-Probe. –No difference was found between the two settings of the PIPS laser (10 mJ vs. 20 mJ).
Effect of different laser-assisted irrigation activation techniques on apical debris extrusion. Doğanay Yıldız et al. (2020) [28].	–Comparison of debris extrusion in four different laser-assisted activation techniques. 60 mandibular premolars were divided equally into four groups: (1) needle irrigation group, (2) photon-induced photoacoustic streaming (PIPS) laser, (3) erbium-doped yttrium aluminum garnet (Er:YAG) laser, (4) neodymium-doped yttrium aluminum garnet (Nd:YAG) laser.	–Conventional needle irrigation provoked significantly less debris extrusion than laser-assisted irrigation activation groups. –Laser-assisted irrigation activation groups produced statistically similar debris extrusion.

Discussion

Based on selected studies, authors have consistently shown interest over the years in assessing the occurrence of apical extrusion during endodontic procedures. This phenomenon has been evaluated using traditional experimental designs such as the Myers and Montgomery setup, as well as modified and innovative methodologies [4]. Each existing research method and experimental model was described in a 2022 review by Tanalp, which summarized their advantages and drawbacks in a critical analysis. The review concluded that no ideal methodology yet exists to meet the requirements of an acceptable extrusion study. It also noted that all the studies presented employed a quantitative evaluation method. Studying the phenomenon solely from a quantitative perspective fails to address it in its entirety. A purely numerical representation of extrusion does not account for biological circumstances, such as content and virulence. A qualitative analysis would offer a more comprehensive approach to clinical projection, taking into account multiple parameters mentioned in the study, such as the physical and chemical characteristics of teeth, the condition of pulpal tissue, the resistance and pressure of periapical tissue, host response, proximity to anatomical structures, and the masticatory characteristics of individuals [4].

Regarding the results of selected studies, apical extrusion appears to be an inherent incident, occurring due to a combination of several factors. The influence of these factors can be analyzed, although it may vary from one study to another.

Influence of Canal Curvature

Most studies on apical extrusion have focused on single-rooted canals with a relatively straight angle (<10°) to study the influence of other factors. However, in clinical conditions, the practitioner is faced with all kinds of canal curvature. To study the influence of canal curvature, Borges et al. in 2011 evaluated extrusion in canals with mild (<10°) and moderate curvature (ranging between 11° and 25°). This comparison proved the absence of any significant difference between the two types of canals when the apical diameter of preparation is at 30.02. However, the amount of extrusion becomes significantly greater in canals with mild curvature compared to the moderate ones when prepared with a 45.02 instrument diameter. These results are explained by the greater alignment between the foramen and the canal direction when canals are mildly curved [5].

Aydin et al. in 2017, quantified bacterial extrusion in three canal morphologies: narrow straight canals, large straight canals, and curved canals (25° to 35°). The results of apically extruded bacteria were statistically similar in all groups. Aydin et al. concluded that effective chemo-mechanical preparation is more important than the morphology of the canal in terms of bacterial extrusion [6].

Influence of Working Length

Many authors have studied the ideal working length to determine prior to instrumentation. This determination should fulfill the objectives of instrumentation without compromising periapical tissue healing. Teixeira et al. evaluated bacterial extrusion for different working lengths and observed no statistically relevant difference. The results revealed no significant difference in bacterial extrusion between foraminal instrumentation and instrumentation 1mm short of the foramen [7].

Recently, in 2019, Mendonça de Moura et al. compared apical extrusion in different canals up to different working lengths. Their findings mirrored those of the previous study. No statistically significant difference was noted regarding the amount of debris extruded. They concluded that the working length had no influence on the amount of extruded debris [8].

Influence of Apical Diameter

The apical preparation of the canal requires the prior determination of two parameters: the working length and the apical diameter. The latter is an important factor that influences the quality of the root canal filling and, therefore, the success of the endodontic therapy. Studies concerning apical diameter as an influencing factor have evaluated different types of extruded content [9,10,11].

In terms of irrigant extrusion, Chang et al. in 2015 asserted that increasing the apical diameter size in preparation decreases the risk of irrigant extrusion. The study explained this by stating that irrigant flow improves in larger-size canals, which reduces the 'back pressure' developed near the tip of the needle [9]. Conversely, in 2016, Silva et al. evaluated the extrusion of debris and demonstrated that larger apical preparation sizes do not influence debris extrusion [10].

Later, in 2021, Cuellar et al. focused on bacterial extrusion by comparing two diameter sizes: 25.06 vs. 30.05. The results were not significantly different for bacterial extrusion regarding the tested file diameters. This study refuted any influence of apical diameter size on bacterial extrusion [11].

In summary, studies are inconclusive regarding the correlation between the quantity of apical extrusion and the apical diameter size. Due to the lack of consensus on the appropriate choice of instrument size among the studies, the importance of apical preparation has been discussed [11]. Some authors have proposed suggestions, such as predetermined sizes beyond 30 or 35 by Siqueira et al., or increased enlargements by three instruments beyond the anatomical diameter by Souza et al. [29,30]. Other authors have mentioned that the instrument taper is a more important factor than its tip size [31].

Influence of Glide Path and Pre-Flaring Procedures

The glide path and pre-flaring techniques are essential preliminary steps for optimizing canal shaping procedures, as they enable sufficient enlargement of the canal before introducing the first shaping instrument [32]. The glide path is the initial step in exploring the canal. Despite the lack of consensus on its definition, it is commonly described as a smooth-walled root tunnel extending from the root canal entrance to its physiological terminus (foraminal constriction). It can be achieved by repeatedly, predictably, and effortlessly passing a manual K-file up to the working length. However, in certain clinical situations, the glide path is not sufficient for the safe passage of rotary instruments. In such cases, it is advisable to add an operative step called 'pre-flaring.' Pre-flaring widens the canal coronally to accommodate the tip of the first shaping instruments. This step was developed to reduce the risk of instrument fracture due to torsion, by reducing the risk of both blocking and tip damage [32].

In terms of extrusion, Tanalp and Güngör in a 2014 review noted that the quantities of extrusion were negligible in these preliminary procedures compared to those recorded during the main shaping procedure. However, since the extrusion during the glide path process occurs first chronologically, the virulence of the micro-organisms within increases. Thus, analyzing their involvement in extrusion is of interest [33].

In 2020, Keskin et al. compared the debris produced during glide path procedures using different instruments. The study concluded that manual glide paths produced significantly more extrusion compared to engine-driven instruments. No statistically relevant differences were recorded between reciprocating motion (R-pilot and WaveOne Gold Glider) and continuous rotational (ProGlider) groups [12].

Topçuoğlu et al. in 2016 evaluated the effect of coronal flaring using Gates-Glidden files during NiTi single-file instrumentation. They concluded that coronal flaring reduced debris extrusion only when canal shaping was performed using reciprocating systems, as opposed to continuous rotation systems where flaring had no effect on debris extrusion. They noted that reciprocating instruments tend to transport debris to the apical region, and coronal flaring creates a reservoir in the coronal third, facilitating evacuation without accumulation in the middle and apical thirds [34]. Gunes and Yeter in 2020 conducted a similar study evaluating the effect of pre-flaring on different NiTi systems. Consistent with the findings of Topçuoğlu et al. in 2016, they found that pre-flaring had no effect on the amount of debris extruded for systems with a continuous rotational dynamic [35].

Influence of Instrumentation Techniques and Systems

Instrumentation systems can be distinguished according to their design, the number of files per system, their preparation technique, and their dynamics.

Instrument design: First of all, there is Karataş et al. in 2016 evaluating apical extrusion while using two systems with gold technology (Protaper Gold and WaveOne Gold). This gold technology is acquired by undergoing heat treatment. Which according to the manufacturer, promises better flexibility and resistance to cyclic fatigue. Karataş et al. concluded that instruments with thermal treatment extruded significantly less debris than instruments with no gold technology [13]. However, Gunes and Yeter in 2020, while evaluating three systems with different cross sections, found no significant difference in terms of debris extrusion. Their study compared Protaper Next, 2Shape, and OneCurve. Note that the cross-section also depends on the heat treatment undergone by the instrument [35].

Number of files per system: Regarding the number of files, Caviedes-Bucheli et al. in 2016 conducted a systematic review and meta-analysis. It was based on nine in vitro studies and two in vivo studies. And it focused on the influence of the number of instruments on the apical extrusion of debris and their biological relationship with the occurrence of symptomatic apical periodontitis. All root canal instrumentation techniques, either reciprocating single-file or rotary-file system, produced apical extrusion of debris or expression of neuropeptides. The meta-analysis stated that the inflammatory reaction is not influenced by the number of files but by the type of movement and instrument design [14]. These results are in discordance with the ones from Sun et al. found in 2018. The meta-analysis concluded a significant difference in extrusion depending on the number of files per system. Accordingly, multiple rotary-file systems contributed to a lower incidence of postoperative pain in comparison to reciprocating single-file systems [15].

Type of instrument: As to the instrumentation type (manual vs. engine-driven), its influence on extrusion was studied by Mittal et al. in 2015. It demonstrated that all tested systems resulted in bacterial extrusion, with the manual step-back technique provoking significantly more than the engine-driven systems. These results were attributed to the linear filing motion and the coronal direction of preparation in the manual technique [16]. In 1995, al Omari and Dummer attested that preparations using a linear filing motion generated more debris than those using a rotational motion [36]. Also Goerig et al. in 1982 evoked that the step-back technique extruded more compared to the crown-down technique [37]. This may conclude that the influence of the instrument type is closely related to the influence of these two parameters: the preparation technique and the instrument dynamics.

Preparation technique: To explain the results obtained by Mittal et al. in 2015, some justifications were established for every direction of instrumentation [16]. During step back, not only the space available for coronal flushing is small, but the file acts as a plunger in the apical third of the canal forcing debris apically [36]. On the other hand, the prior preparation of the coronal section during crown down helps in reducing the amount of microorganisms and contaminants that could be pushed during apical instrumentation [37]; since the greatest amount of microorganisms present in a canal is in its coronal third [38]. Additionally, the manual control of the operator on instruments while preparing the apical third is also improved due to the coronal preflaring during crown down [37].

Instrument dynamic: In parallel to the preparation technique, Mittal et al. implicate the instrument dynamic as an influencing factor too [16]. The association of the linear motion of translation to significantly more debris extrusion than rotational motion techniques was confirmed by al-Omari and Dummer [36]. It may be explained as follows: for linear filing motion, files may act as pistons pushing irrigating solutions and debris toward the apex [39]. And concerning rotational motion whether in instrumentation with engine-driven or balanced force techniques: a collection of debris is formed into the flutes of the instrument and is evacuated out of the root canal in a coronal direction [40,41]. These hypotheses may justify the increasingly popular use of the crown-down technique, given the numerous disadvantages of linear motion in the step-back technique.

On the other hand, it is also necessary to mention that within the rotational motion, there are different dynamics including reciprocity and full rotational motion (continuous rotation). These two dynamics in particular were evaluated in several studies through the years. The common point between all the studies comparing the two dynamics is their agreement on the undeniable nature of the extrusion phenomenon. All studies stated that all systems, whatever their instrumental dynamics might be, caused apical extrusion to some degree. And none of them was spared from this predicament [17,18,19,20,21,42].

The results of certain studies confirmed the absence of difference between the two dynamics [17,18,19]. Such as Küçükyilmaz et al. in 2015, who mentioned the significantly equal amount of debris and irrigant extruded for all the dynamics tested [17]. Similar results were obtained in a study by Al Omari et al. in 2023, where no statistically significant difference emerged between continuous rotation and reciprocation motion [18]. Comparable results were also found in a prospective randomized clinical trial by Kumar et al. in 2023, aiming to compare the incidence of postoperative pain and intake of analgesic medication after endodontic treatment using different systems. It actually concluded no statistically significant difference among the two dynamics regarding the incidence of postoperative pain [19].

Some authors like Uzun et al. in 2016 compared extrusion during canal shaping in 60 canals and using three continuous rotation systems (Typhoon, Protaper Universal, Mtwo) to three reciprocating ones (WaveOne, Reciproc, SafeSider). The study attested only one statistically significant difference between the Reciproc system and the Typhoon system with continuous rotation. In which the Reciproc system generated less extrusion. The extrusion caused by the rest of the systems was significantly equal [42]. In addition, interesting findings were made in 2018 by Alves et al. attesting that similar amounts of hard tissue debris were produced in both techniques. However, bacterial extrusion was significantly lower in reciprocity compared to continuous rotation [20]. A more recent review by Ahmad et al. from 2022 compared four different single-file instrumentation systems. It included studies comparing reciprocating systems (WaveOne / Reciproc) to full rotational systems (NeoNiTi / OneShape). From the 124 articles found eligible only eight studies were included in the meta-analysis, the authors also declared that the overall risk of bias was too low. This review states that root canal preparation with continuous motion provokes a lower amount of apically extruded debris compared with reciprocating instrumentation [21].

Influence of Irrigation Procedures

In parallel to preparation systems, irrigation is also a determining factor in effective chemomechanical preparation [43]. Type of root canal irrigant: The use of intracanal irrigation solutions is intended to disrupt the biofilms that adhere to the surfaces of the canal, dissolve, and eliminate all debris and microorganisms [43]. However, this action must remain effective without harming the health of the peri-apical tissues, which implies the importance of studying its influence on apical extrusion. A study carried out in 2018 by Barbosa-Ribeiro et al., compared four different irrigating solutions: 6% sodium hypochlorite (NaOCl), 2% chlorhexidine gel + saline solution (CHXg + SS), 2% chlorhexidine solution (CHXs), and saline solution (SS), using positive or negative pressure: conventional irrigation by syringe or Endovac. The study concluded that during irrigation by Endovac, no difference was significantly relevant among the irrigants. However, while using conventional irrigation, 2% chlorhexidine gel + saline solution (CHXg + SS), extruded significantly less debris [22]. 

These results were in concordance with the ones concluded in a later study by Arruda-Vasconcelos et al. in 2019. The study explained the results by the viscosity and rheological action of Chlorhexidine in a gel formulation, which keeps debris in suspension during conventional irrigation. Also, the absence of difference between tested irrigants during Endovac might suggest that mechanical action is the main responsible for preventing extrusion [44].

Irrigant needle design: Other studies have focused on needle design as an influencing factor in apical extrusion. Psimma et al. in 2013 quantified apical extrusion of irrigant delivered by an open-ended needle and closed-ended needle. The results were that open-ended needles caused significantly more extrusion compared to closed-ended needles. This can be explained by the intense jet produced and extended far beyond its exit when using open-ended needles. Making the jet difficult to control clinically, even if it is very interesting in terms of fluid dynamics. Contrary to closed-ended needles, the jet obtained is very different because it extends to a very limited extent beyond the end of the needle, regardless of the design of the closed-ended needle used. Which guarantees the safety of use. Also, the jet produced bypasses the end of the needle and is mainly directed towards the canal wall. This allows the backflow of irrigant, causing more coronal displacement of debris while avoiding accidental transfer into the periapex [23].

Yeter et al. conducted the same experiment and justified its similar results by the difference in irrigant pressure at the foramen between the two types of needles. Yeter et al. analyzed the pressure at the foramen by a computational fluid mechanics model (CFD). At the same depth in the canal, an open-ended needle leads to a higher pressure in the apical region than a closed-ended one. Moreover, Yeter et al. added that the open-ended needle produced an additional 1mm irrigant flow toward the apex [24].

Needle insertion depth: Psimma et al. also studied insertion depth as an influencing factor, and compared different needle insertion depths at 1mm, 3mm, and 5mm. Irrigant extrusion decreased as the needle moved away from the working length. In general, the study concluded that positioning the needle farther away from the working length has been associated with reduced irrigant extrusion. Irrigant pressure at the foramen has also been reported to decrease [23].

Injection rate: As to the injection rate, it was studied by Chang et al. in 2015 [9]. According to this study, flow rates in manual irrigation while in a clinical situation are reported to be ranging between 30 and 320 μl/s. With an average of 137 μl/s for male operators and 88 μl/s for female operators. These numbers were exposed as a means of comparison since the flow cannot be controlled in manual irrigation. The experiment was conducted using a syringe pump with controllable flow and evaluated four rates: 50, 100, 200, and 300 μl/s. Also, the manual syringe was moved back and forth throughout the experiment to avoid intracanal blockage, unlike the pump which remained stationary [9]. 

Chang et al. declared that extrusion affected all groups regardless of injection rate, the difference being in the degree of extrusion. In general, the extrusion is greater during manual irrigation and is explained by the back-and-forth movement, in particular during the descent of the needle towards the apex. Which momentarily increases the apical hydraulic pressure because of the reduced space in front of the needle. This increased pressure is actually the driving force leading to irrigant leakage through the apical foramen [9]. For the syringe pump irrigation: no difference of extrusion is observed when the flow rate is less or equal to 100 μl/s. Therefore, no influence on the extrusion is noted. Yet, starting from 200 μl/s, the flow began to play an important role in extrusion. The study recognizes that increasing the injection rate above 200 μl/s increases the risk of irrigant extrusion [9]. Irrigant delivery system: Even if irrigation is most commonly delivered in the endodontic system by the conventional syringe using positive pressure. A commercially available irrigation device was developed to improve irrigant delivery. This system uses apical negative pressure (ANP), where the direction of irrigation is reversed. The solution is in fact brought into the access cavity and then is aspirated towards the apical zone by a micro-cannula connected to the suction. Romualdo et al. in 2017 aimed in their systematic review and meta-analysis to assess if apical negative pressure (ANP) prevents the apical extrusion of debris and irrigant. All studies included evaluated the Endovac system. Romualdo et al showed that ANP prevents the apical extrusion of irrigant. Although, there is no evidence that this type of irrigation prevents the extrusion of debris [25]. In 2018, Barbosa-Ribeiro et al. tested the Endovac system as well. The study also confirmed that the ANP system provides undeniable irrigation safety through lower extrusion, without ensuring zero debris extrusion [22]. 

Irrigant activation system: There are some devices on the market to activate the irrigating solution within the canal. Their purpose is to improve irrigation through the canal network while promoting the effective renewal of its irrigant. It is therefore evident to study the safety and influence of these devices on apical extrusion. Among the most popular modes of activation, there is ultrasonic activation also called passive ultrasonic irrigation (PUI).

Cuellar et al. in 2021, evaluated its influence on bacterial and debris extrusion in comparison to irrigation with no activation. The results indicate that apical extrusion occurs in all the techniques evaluated. However, in the absence of activation, less extrusion of debris and bacteria is produced compared to passive ultrasonic irrigation [11]. Ultrasonic agitation in the apical third of the canal could thereby force solution towards the foramen which then increases the extrusion of debris. This would result in greater bacterial extrusion. These results do not underestimate the major role ultrasonic activation can have in improving the asepsis of the canal. It remains strongly recommended especially for areas like the isthmus where *Enterococcus faecalis* and other species can remain viable as is the case for persistent infections [11]. Activation of irrigants can also be achieved through other modes. Azim et al. in 2018 tested three kinds of activation: the mechanical activation by XP-Endo Finisher, the sonic activation by EndoActivator, and the laser activation by photo-induced photoacoustic streaming (PIPS). EndoActivator and XP-Endo Finisher presented similar extrusion volumes. While PIPS laser activation had a significantly higher extrusion volume than all the other modes, due to the energy produced by the Er:YAG laser. It also seemed unrealistic for the authors to consider the possibility that no drop of irrigating solution could penetrate the periapex [26].

Actually, Yost et al. 2015 have recorded, using a high-definition camera, microbubbles coming out of the apex when laser activation by PIPS is used. Even after reducing the power settings of the laser and moving the insert away, by placing it in the pulp chamber, nothing seemed to diminish the extrusion potential of this method of activation [27]. 

Other types of lasers can be used to activate the irrigating solutions, they were compared in a study by Doğanay Yıldız et al. in 2020. The comparison concerned laser by PIPS, erbium-doped yttrium aluminum garnet (Er:YAG) laser, and neodymium-doped yttrium aluminum garnet (Nd:YAG) laser. The results agreed that irrigation with laser activation produced more apical extrusion than irrigation without activation. Also, no statistically significant difference was relevant between the three lasers [28].

Prevention and guidelines

Following the various factors presented previously and their influence on apical extrusion, it appears that its occurrence is undeniable and cannot be avoided. The results of all studies are quite explicit on the inevitable nature of this phenomenon. However, we also understand that it can be decreased significantly by modifying certain parameters and preferring certain techniques, despite the inherent nature of extrusion. Therefore, preventing extrusion can only be achieved by acting on the quantitative parameter. As a consequence, instead of avoiding the extrusion as a whole, the prevention would be oriented towards a lesser apical extrusion. A number of recommendations have been drawn, they are to consider for every endodontic treatment and not only to reduce the amount of extrusion as much as possible: (1) establish a precise diagnosis before any endodontic treatment [45]; (2) follow strict guidelines concerning asepsis/antisepsis during the process by placing a rubber dam which remains obligatory to isolate the treated tooth while controlling cross-infections and ensuring the removal of all pulp chamber contents, including carious tissue and all the potentially contaminated tissue or material [45]; (3) determinate the appropriate apical working length without violating the apical constriction: the working length should be as close as possible to the physiological apex while staying limited to the canal content [46]; (4) consider the importance of glide path and pre-flaring procedures in facilitating the use of the shaping instruments up to the working length [32]; (5) constant respect for the chosen working length for all instruments; (6) preserve the position and diameter of the apical foramen as well as its apical constriction; (7) maintain permeability throughout shaping using the "apical patency" technique without compromising the apical constriction; (8) clean the flutes of each file after each instrumental passage; and (9) irrigate after each instrumental passage. 

More specifically regarding apical extrusion, the following preventive measures can be concluded: For precautions related to root canal instrumentation, when choosing NiTi alloy instruments, it is advised to prefer heat-treated ones and favor the crown-down technique. As for measures concerning canal irrigation, it is preferable to choose closed-ended needles over open-ended ones [45], ensure that the irrigation needle is loosely inserted and placed away from the apical region [45] and inject using light pressure while avoiding back-and-forth movements along the canal [45]. It is also beneficial to favor the use of apical negative pressure when possible, such as with Endovac [11]. If a practitioner is hesitant about the use of passive ultrasonic irrigation, two pieces of advice are suggested: first, use ultrasonic activation only after chemo-mechanical decontamination to avoid any possible extrusion of contaminated debris; and second, place the ultrasonic insert at 3 mm or more short of the working length [11].

## Conclusions

Despite the clear role of different factors presented, such as instrumentation techniques, instrument design, irrigation methods, and dynamics, the occurrence of apical extrusion during endodontic procedures is undeniable. The discordant results between different studies may look confusing for readers, which can be essentially due to the experimental methods used. Providing experimental models that approach in vivo conditions appears to be necessary to establish exploitable conclusions. Also, creating a unified model and method to assess extrusion might be the solution for confronting study outcomes. Even if extrusion cannot be avoided completely, practitioners can reduce its quantity by considering proposed recommendations, which can therefore reduce its clinical complications.
